# Cross-sectional Serosurvey of Crimean-Congo Hemorrhagic Fever Virus IgG in Livestock, India, 2013–2014

**DOI:** 10.3201/eid2110.141961

**Published:** 2015-10

**Authors:** Devendra T. Mourya, Pragya D. Yadav, Anita M. Shete, Padmakar S. Sathe, Prasad C. Sarkale, Bramhadev Pattnaik, Gaurav Sharma, Kamlesh J. Upadhyay, Surekha Gosavi, Deepak Y. Patil, Gouri Y. Chaubal, Triparna D. Majumdar, Vishwa M. Katoch

**Affiliations:** National Institute of Virology, Pune, India (D.T. Mourya, P.D. Yadav, A.M. Shete, P.S. Sathe, P.C. Sarkale, S. Gosavi, D.Y. Patil, G.Y. Chaubal, T.D. Majumdar);; Project Directorate on Foot-and-Mouth Disease, Mukteswar, India (B. Pattnaik, G. Sharma);; B.J. Medical Colleges, Ahmedabad, India (K.J. Upadhyay);; Indian Council of Medical Research, Ministry of Health & Family Welfare, Ansari Nagar, India (V.M. Katoch)

**Keywords:** Crimean-Congo hemorrhagic fever, ticks, viruses, IgG, antibodies, livestock, serosurvey, Gujarat State, India, zoonoses, vector-borne infections, sheep, goats, cattle, bovines, bovids

## Abstract

We conducted a cross-sectional serosurvey of Crimean-Congo hemorrhagic fever (CCHF) among livestock in 22 states and 1 union territory of India. A total of 5,636 samples from bovines, sheep, and goats were screened for CCHF virus IgG. IgG was detected in 354 samples, indicating that this virus is widespread in this country.

Crimean-Congo hemorrhagic fever (CCHF) is caused by a virus (CCHFV) that belongs to the family *Bunyaviridae*, genus *Nairovirus* (*1**,**2*)*.* CCHF causes severe illness in humans and has a case-fatality rate of up to 80% (*3**,**4*)*.* The disease is widespread in various countries in Africa, Asia, southeastern Europe, and Eurasia, and cases have been documented recently in India (*4**–**7*). The virus is transmitted to humans when they are bitten by *Hyalomma* spp. ticks, which are usually found on cattle, buffalo, goats, and sheep. Humans can also be infected through nosocomial transmission or from the blood, tissues, or bodily secretions of an infected animal when it is slaughtered or during related procedures (*1**,**4**,**8**,**9*)*.*

The first confirmed cases of CCHF in India occurred during a nosocomial outbreak in Ahmadabad, Gujarat, in January 2011 (*9*)*.* During 2012–2015, several outbreaks and cases of CCHF transmitted by ticks via livestock and several nosocomial infections were reported in the states of Gujarat and Rajasthan. Cases were documented from 6 districts of Gujarat (Ahmadabad, Amreli, Patan, Surendranagar, Kutch, and Aravalli) and 3 districts of Rajasthan (Sirohi, Jodhpur, and Jaisalmer) (*10**,**11*). Recently, a CCHF case was reported from Uttar Pradesh state (http://promedmail.chip.org/pipermail/promed-soas/2015-February/000582.html).

In the past, serologic evidence of CCHFV infection was reported in India from animal serum samples collected in western border districts, southern regions, Maharashtra state, and Jammu and Kashmir state (*12*)*.* A recent serosurvey conducted in 15 districts of Gujarat revealed the presence of CCHFV IgG in a substantial proportion of domestic animals (*13*)*.* On the basis of these data, we conducted a countrywide cross-sectional serosurvey of livestock to determine the presence of CCHFV in India.

## The Study

Working with the Indian Council of Agricultural Research, we asked foot and mouth disease (FMD) centers throughout India to send us serum samples from bovines, goats, and sheep. We requested >200 representative samples from each state and only used those that tested negative for FMD. The number of samples varied (99–357 for bovine samples and 19–260 for sheep and goat samples), depending on where the samples were collected and the population of each animal in that area.

We detected CCHFV-specific IgG in the serum samples by using 2 ELISA kits (1 for bovines and 1 for sheep and goats) that were developed by the National Institute of Virology (NIV) in Pune, India. We coated Nunc MaxiSorp plates (Thermo Fisher Scientific, Waltham, MA, USA) with γ-inactivated CCHFV (positive antigen) and negative control tissue culture fluid (negative antigen) diluted in carbonate buffer and incubated them overnight at 4°C. Plates were washed 3 times with 1× phosphate-buffered saline with 1% Tween-20 (PBST) and further treated with postcoating buffer. Plates were washed then 3 times with 1× PBST. Serum samples were diluted in sample dilution buffer (1:200 dilutions for bovine samples and 1:2,000 dilutions for sheep/goat samples). Positive and negative control animal serum samples were included in triplicate for each assay by using similar dilution for quality control.

Samples were added to both the positive and negative antigen-coated rows and incubated at 37°C for 45 min. After washing the plates 5 times with 1× PBST, we probed the wells with bovine or sheep IgG conjugated with biotin for the respective ELISAs and incubated the plates for 1 hour. We washed the plates 5 times with 1× PBST, incubated them with avidin-horseradish peroxidase for 30 min at 37°C, then washed them 5 times with 1× PBST. We added 3,3′,5,5′-tetramethylbenzidine substrate and incubated the plates for 10 min in the dark at room temperature; the reaction was stopped by using 1N H_2_SO_4._ Finally, we read the plates with a spectrophotometer at 450 nm. The ratio of optical density of positive and negative controls was taken for each sample (P/N ratio). The sample was considered positive when the P/N ratio was >1.5 from both kits (*14*).

The sensitivity and specificity of these kits were tested and compared with known standards by using ELISA reagents provided by the Centers for Disease Control and Prevention (Atlanta, GA, USA). Bovine ELISA showed 80.5% sensitivity and 96.05% specificity, and sheep/goat ELISA showed 63.6% sensitivity and 100.0% specificity. The performance of these kits was validated by 3 NIV laboratories and 3 other national laboratories in India.

We screened 5,636 (4,781 bovine and 855 sheep and goat) animal serum samples from 22 states and 1 union territory for CCHFV IgG; samples were not obtained from 7 states and 6 union territories. Only 8 states and 1 union territory provided sheep and goat samples. Overall, 260 (5.43%) of 4,781 bovine samples and 94 (10.99%) of 855 sheep/goat samples tested positive for CCHFV IgG (Table; Figure). Among bovine samples, maximum IgG positivity was seen in Orissa (31.3%); for sheep and goat samples, maximum IgG positivity was seen in Himachal Pradesh (53.1%) (Table). Seropositivity levels found in these animals suggested prevalence of CCHFV infection among livestock in these states across the country.

**Table:** CCHFV IgG positivity detected in serum samples from bovines, sheep, and goats from 22 states and 1 union territory, India*

**Location of center**	**No. positive samples/no. samples tested (%)**	
	Bovine	Sheep and goat
**Andhra Pradesh**	30/233 (12.9)	−
**Madhya Pradesh**	7/150 (4.7)	3/40 (7.5)
**Maharashtra**	16/240 (6.7)	–
**Punjab**	6/198 (3.0)	–
**Rajasthan**	31/295 (10.5)	–
**Orissa**	31/99 (31.3)	–
**Arunachal Pradesh**	13/150 (8.7)	5/71 (7.0)
**Karnataka**	15/219 (6.8)	–
**Kerala**	5/200 (2.5)	5/219 (2.3)
**West Bengal**	13/357 (3.6)	–
**Manipur**	1/146 (0.7)	0/49
**Mizoram**	15/203 (7.4)	–
**Tamil Nadu**	6/379 (1.6)	–
**Uttar Pradesh**	7/195 (3.6)	–
**Tripura**	1/149 (0.7)	–
**Guwahati**	26/149 (17.0)	0/50
**Haryana**	3/200 (1.5)	–
**Himachal Pradesh**	1/150 (0.7)	26/49 (53.1)
**Jammu and Kashmir**	4/182 (2.2)	0/19
**Nagaland**	8/199 (4.0)	–
**Andaman and Nicobar**	2/308 (0.7)	21/98 (21.4)
**Uttarakhand**	2/100 (2.0)	–
**Gujarat**	17/280 (6.1)	34/260 (13.1)
**Total**	260/4,781 (5.43)	94/855 (10.99)

*CCHFV, Crimean-Congo hemorrhagic fever virus; FMD, foot and mouth disease; −, no samples available for screening.

**Figure:**
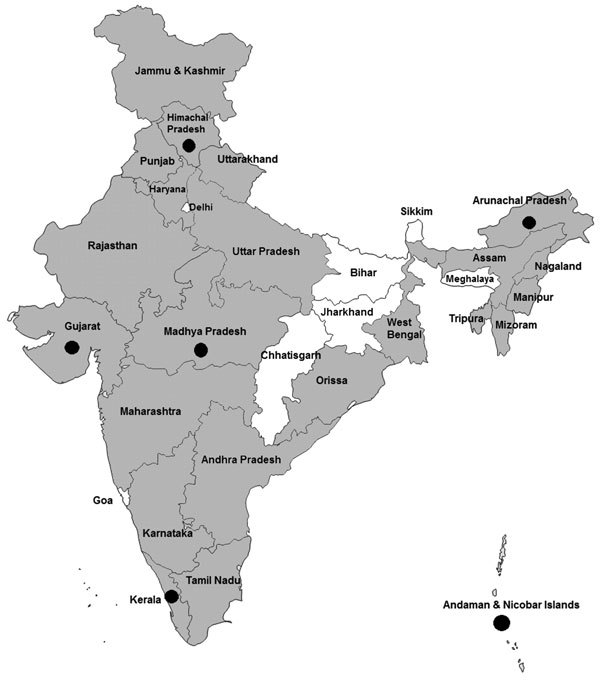
Location of Crimean-Congo hemorrhagic fever virus IgG seropositivity in bovines, sheep, and goats in 22 states and 1 union territory, India. Gray shading, seropositivity in bovines; black dots, seropositivity in sheep/goats; white, serum samples not available screening.

## Conclusions

A large portion of the economy of India and the country’s rural development depend on agriculture, livestock farming, and the dairy industry (http://poshan.nic.in/jspui/bitstream/DL/1247/1/nfi_01_00_643.pdf, http://dahd.nic.in/dahd/updates/whats-new/18th-livestock-census-2007.aspx)*.* Because India hosts many animal trading fairs each year (e.g., Pushkar fair, Uttar Pradesh; Sonepur Animal Mela, Bihar), tick-infested animals move throughout the country. India also exports >US$400 million of meat. Such widespread animal trade and exports can pose a high threat of transmission of pathogens such as CCHFV to newer areas. The country experienced similar situations during suspected plague outbreaks and outbreaks of infection with avian influenza, which not only resulted in considerable economic losses but also created panic in the community. Although our survey showed scattered geographic distribution of CCHFV IgG among livestock in India, data from 5 years of investigations in Gujarat suggest that active surveillance in any of these states would probably reveal a more accurate estimate of CCHF prevalence.

This study suggests that animal husbandry and abattoir workers are at high risk because they are always in close contact with livestock or carcasses that may be infested with CCHFV-infected ticks (Table; Figure). Because viremia in livestock is short-lived (up to 2 weeks) and of low intensity, infected animals do not develop severe disease, but they may still transmit the virus to other animals and to humans.

Diagnosis of high-risk group pathogens is a major concern in India, where few Biosafety Level 3 laboratories and only 1 Biosafety Level 4 laboratory exist. Therefore, there is also a need to make available safe diagnostic tests that can be used at primary health centers, medical colleges, and all other health settings across the country. The CCHFV IgG ELISA kits developed by NIV could help in monitoring CCHFV prevalence and the findings could make it possible for public health authorities to develop proactive preparedness programs that would enable them to send alerts and develop precautionary measures.
